# Fear Learning Increases the Number of Polyribosomes Associated with Excitatory and Inhibitory Synapses in the Barrel Cortex

**DOI:** 10.1371/journal.pone.0054301

**Published:** 2013-02-14

**Authors:** Malgorzata Jasinska, Ewa Siucinska, Ewa Jasek, Jan A. Litwin, Elzbieta Pyza, Malgorzata Kossut

**Affiliations:** 1 Department of Histology, Jagiellonian University Medical College, Krakow, Poland; 2 Department of Molecular and Cellular Neurobiology, Nencki Institute of Experimental Biology, Warsaw, Poland; 3 Department of Cell Biology and Imaging, Institute of Zoology, Jagiellonian University, Krakow, Poland; 4 Warsaw School of Social Psychology, Warsaw, Poland; CNRS UMR7275, France

## Abstract

Associative fear learning, resulting from whisker stimulation paired with application of a mild electric shock to the tail in a classical conditioning paradigm, changes the motor behavior of mice and modifies the cortical functional representation of sensory receptors involved in the conditioning. It also induces the formation of new inhibitory synapses on double-synapse spines of the cognate barrel hollows. We studied density and distribution of polyribosomes, the putative structural markers of enhanced synaptic activation, following conditioning. By analyzing serial sections of the barrel cortex by electron microscopy and stereology, we found that the density of polyribosomes was significantly increased in dendrites of the barrel activated during conditioning. The results revealed fear learning-induced increase in the density of polyribosomes associated with both excitatory and inhibitory synapses located on dendritic spines (in both single- and double-synapse spines) and only with the inhibitory synapses located on dendritic shafts. This effect was accompanied by a significant increase in the postsynaptic density area of the excitatory synapses on single-synapse spines and of the inhibitory synapses on double-synapse spines containing polyribosomes. The present results show that associative fear learning not only induces inhibitory synaptogenesis, as demonstrated in the previous studies, but also stimulates local protein synthesis and produces modifications of the synapses that indicate their potentiation.

## Introduction

Sensory experience and learning can induce changes in the connections between neurons. Alterations of synaptic and spine density were observed in several learning paradigms [1], [2], [3], [4], [5], [6] as well as in case of enhanced or decreased sensory stimulation [7], [8], [9], [10], [11]. Modifications of the neuronal activity have also been shown to change synaptic efficiency by inducing a remodeling of the existing synapses [12], [13], [14].

We have investigated microstructure of somatosensory barrel cortex following fear learning-induced plastic change. Short-lasting classical conditioning, in which stimulation of a row of whiskers is paired with the administration of a mild electric shock to the tail, produces plastic modification in layer IV of the barrel cortex. This part of somatosensory cortex offers an excellent model to study the mechanisms of learning and memory because of its clearly defined structure, and the ease of inducing plasticity via a fear learning paradigm [15], [16]. 2-deoxyglucose autoradiography revealed enlargement of functional cortical representation of the whiskers stimulated during conditioning [16]. We have shown that this conditioning paradigm changes motor behavior of mice by decreasing the number of head movements in response to the conditioned stimulus (a phenomenon akin to freezing observed in auditory fear conditioning) [17]. It is also associated with an autonomic response, conditioned bradycardia [16]. The learning-induced plastic change of cortical representation is associated with a number of alterations in the inhibitory neurotransmission. It is paralleled by a rapid increase of GAD67 mRNA, and increased density of GAD and GABA immunoreactive cells in the barrels of the row receiving input from the stimulated whiskers [18], [19]. Conditioning led to the enhancement of inhibitory synaptogenesis [20], as well as to increased inhibitory transmission [21] in layer IV of the “trained” barrel. It also affected excitatory transmission by changing subunit composition of NMDA receptors. Increase in mRNA and protein of NMDA2A subunit was found in the barrels receiving the conditioned stimulus [22].

The presence of polyribosomes near the synapses in the dendritic spines or shafts suggests the involvement of local protein synthesis in the process of synapse modification [12], [23], [24]. The newly synthesized protein molecules enhance the level of the existing proteins or replace them [12], [25]. Thus, an increase in the number of polyribosomes near the synapses might indicate either a formation of new synapses or a modification of the existing ones as a result of neuronal activity [14].

In the present study, the polyribosomes in the barrel cortex were investigated following the conditioning, by electron microscopy of serial sections and 3D reconstruction of dendritic spines. We found that classical fear conditioning resulted in an increased density of polyribosomes in dendritic spines and shafts associated with inhibitory and excitatory synapses in layer IV barrels receiving the conditioned stimulus. We also observed conditioning-induced enlargement of post-synaptic density (PSD) area of the excitatory and inhibitory synapses on spines containing polyribosomes, which might serve to increase the strenght of synapses.

## Materials and Methods

We examined the same collection of ultrathin sections which was used in our previous study [20].

### Animals

As described previously [20] the experiments were performed on Swiss Webster mice aged 6–7 weeks, raised in standard conditions. All experiments were compliant with the European Communities Council Directive of 24 November 1986 (86/609/EEC) and were approved by the Animal Care and Use Committees of the Polish Academy of Sciences and the Jagiellonian University.

### Behavioral training

The mice (N = 21) were divided into a trained group (N = 14) and a control group (N = 7). The trained mice were either conditioned (N = 7) or pseudoconditioned (N = 7). All animals were habituated in a homemade restrainer which held the mouse neck stationary, leaving the rest of the body, including the head, free. During the habituation period, the mice spent in the restrainer 10 min per day for 3 weeks.

After habituation, the mice from the conditioned group were trained using the classical conditioning paradigm, exactly as described in [16]. Manual stimulation of the selected whiskers (B row; conditioned stimulus, CS) on one side of the snout was paired with a mild electric shock (unconditioned stimulus, UCS) [16]. The pairing procedure included three sweeps along the entire whisker row with a small paintbrush lasting 9 s (CS) and at the last 0.5 s of CS a tail shock (UCS) of 0.5 mA was applied. The CS+UCS pairings were repeated, four times per minute for 10 min. The conditioning sessions were carried out for 3 consecutive days. In the pseudoconditioned animals (random pairing of CS and UCS), the number and frequency of stimuli applied were the same. The behavior of animals was filmed and their head movements in response to CS were analyzed.

### Transmission electron microscopy

Twenty-four hours after the completion of the training, the mice were anesthetized with Vetbutal (25–30 mg/kg body weight; Biowet Pulawy) and perfused through the heart with 20 ml of rinse buffer (0.2% glutaraldehyde and 2% paraformaldehyde in 0.1 M phosphate buffer, pH 7.4) followed by 100–150 ml of fixative (2.5% glutaraldehyde and 2% paraformaldehyde in 0.1 M phosphate buffer, pH 7.4). The brains were removed immediately after perfusion and were left in the same fixative for 24 h at 4°C.

Next, after washing in 0.1 M phosphate buffer (pH 7.4), 60 μm tangential vibratome sections were cut from the barrel cortex. Sections were examined under a stereomicroscope (Nikon Optiphot) and those containing the barrel field cortex were collected for further processing. The sections were washed in 0.1 M cacodylate buffer (pH 7.4), postfixed with 1% osmium tetroxide in 0.1 M cacodylate buffer (pH 7.4) twice (the first time with 1.5% potassium ferrocyanide), washed in 70% ethanol containing 1% uranyl acetate, and after dehydration in a graded series of ethanol, embedded in Epon resin (Polysciences) between two silicon-coated glass slides.

The region of B2 and B3 barrels was identified according to the procedure described previously [20] and trimmed for ultrathin sectioning. A series of 30 to 50 successive sections (60–70 nm thick) were cut from each sample. The sections were collected on formvar-coated copper-palladium slots and contrasted with 1% lead citrate. For an examination of polyribosome density, 6–8 series, consisting of 3 serial electron micrographs each obtained from successive sections containing the B2 barrel central area in which cell bodies are sparse, in were taken at 14K under JEOL 100SX transmission electron microscope (JEOL, Japan). Ten to twelve serial electron micrographs were taken from successive sections for a 3D reconstruction of the dendritic spines. The micrographs were initially aligned in Adobe Photoshop CS software, in which stacks of serial images were taken at the final magnification of 30 K.

### Quantitative analysis of polyribosomes

The quantitative analysis of the polyribosomes was carried out using NIH Image J Cell Counter software (http://rsb.info.nih.gov/ij/) by placing a grid of a two-dimensional sampling frame over the stack of serial sections. The polyribosomes were counted per area unit (μm^2^), because their size usually did not exceed the thickness of a single ultrathin section. Each polyribosome was counted only once in the stack and only the polyribosomes located fully within the frame or intersecting the left and the upper borderlines of the frame were included. Synapses and spines were defined according to [8]. The accordance of synapse morphology (asymmetric/symmetric) with the character of synapses (excitatory/inhibitory) was previously confirmed by immunocytochemistry of presynaptic markers in the same material [20]. Astrocytic processes were distinguished from dendrites according to [26]. Since polyribosomes located in cell bodies were omitted from the analysis, the total density of polyribosomes reflects the number of polyribosomes in dendritic spines and shafts counted in a defined area of B2 barrel. If the polyribosomes were located under the synaptic profiles without any intervening organelles and within the distance of ≤2 μm as measured from the center of the synapse, they were considered as synapse-associated [14], [27].

The density of polyribosomes associated with excitatory and inhibitory synapses and not associated with synapses, located in dendritic shafts and dendritic spines was calculated according to the stereological formula N_A_  =  ΣQ^−^/A, where ΣQ^−^ is the number of polyribosomes counted in the entire area A [28]. The counting was done blind – the observer did not know whether the micrographs were taken from conditioned, pseudoconditioned or control animals.

### Morphological analysis of single- and double-synapse spines

Images of 37 single-synapse spines and 41 double-synapse spines from the control, the conditioned group and the pseudoconditioned group were selected for PSD area measurements and 3D reconstruction. Obliquely or longitudinally sectioned dendritic spines were not taken into consideration. To assess PSD area, three area measurements from each micrograph containing profiles of the selected spines were made using NIH Image J software. 3D reconstructions of the spines were performed using 3D Studio Max software (Discreet Logic, Montreal, Canada) and the location of polyribosomes in the dendritic spine areas (head, neck or base) was estimated.

### Statistical analysis

All the data were analysed using GraphPad Prism 4.0 software (GraphPad Software Inc., USA).

To compare the effects of conditioning on polyribosome density across the experimental groups, one-way ANOVA with *post hoc* Tukey test was used after pretesting for normality and homogeneity. If the absence of Gaussian distribution was noted (D'Agostino and Pearson omnibus normality test) or if variances were significantly different (Bartlett's test for equal variances), the data were analysed by use of the Kruskal-Wallis test with *post hoc* Dunn's test. Differences in the location of polyribosomes in dendritic spines between the control, the conditioned and the pseudoconditioned group were compared by chi-square test. Two-way ANOVA test was used to analyse the combined effects of the training and of the polyribosome distribution on PSD area.

## Results

In this study, we identified the synapses in B2 barrel of mouse somatosensory cortex and divided them according to their location into synapses on dendritic shafts and on dendritic spines. The synapses placed on dendritic shafts were either symmetric (presumptive inhibitory) or asymmetric (presumptive excitatory). All synapses located on single-synapse spines were excitatory, whereas double-synapse spines always had one excitatory and one inhibitory synapse, as demonstrated in earlier studies [8], [29]. We have not found spines with more than two synapses.

### Behavioral effects

The behavioral effects of conditioning in mice used in this experiment were described previously [20]. We examined CS-linked changes in head turning towards the stimulating brush. We considered them as a marker of acquiring a predictive value by the CS. We found that the conditioned mice showed a significant decrease (54+/−2.1%, p<0.05, Mann Whitney test) in head mobility, that developed during the process of conditioning in response to conditioned stimulus (“minifreezing”, [17]). No decrease of head mobility was found in the pseudoconditioned group (109+/−8.9%).

### Sampling areas

Polyribosomes were counted in the following total tissue areas: 2510.06±167.34 μm^2^ (mean area per animal 358.58±61.73 μm^2^) in the control group; 2210.07±147.34 μm^2^ (mean area per animal 315.72±29.78 μm^2^) in the conditioned group; 2147.16±143.14 μm^2^ (mean area per animal 306.74±23.60 μm^2^) in the pseudoconditioned group. These sampling areas were not significantly different across the treatment groups (one-way ANOVA, p = 0.0714, F = 3.067, N = 21, total df  = 20).

### Density of polyribosomes

**Figure 1:**
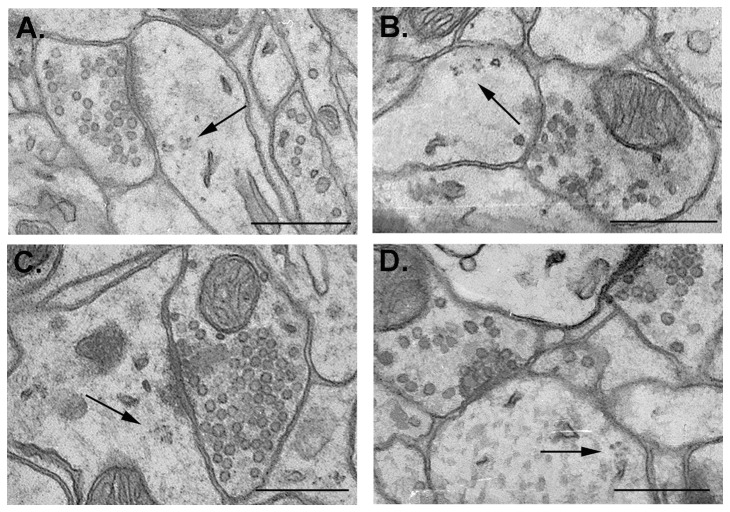
Location of polyribosomes in dendrites of B2 barrel. Electron micrographs of B2 barrel hollow showing polyribosomes (arrows) located in the dendritic spine and associated with excitatory synapse (**A**), located in the dendritic spine and associated with inhibitory synapse (**B**), located in the dendritic shaft and associated with excitatory synapse (**C**) and located in the dendritic shaft and associated with inhibitory synapse (**D**). Scale bar: 0.5 μm.

Individual ribosomes were identified as small (18–25 nm) structures with round and opaque centers surrounded by lighter edges [23], [25]. Three or more ribosomes forming a spiral or an irregular cluster were identified as polyribosome. Polyribosomes were classified according to their location in dendritic spines (Fig. 1A, B) or shafts (Fig. 1C, D).

#### Polyribosomes associated with synapses

The distances between synapse-associated polyribosomes and synapses were not significantly different across the groups (asymmetric synapses: control group 0.045–0.895 μm, conditioned group 0.055–0.940 μm, pseudoconditioned group 0.038–0.852 μm; one-way ANOVA, p = 0.8956, F = 0.1103, N = 21, total df  = 20; symmetric synapses: control group 0.047–0.699 μm, conditioned group 0.025–0.936 μm, pseudoconditioned group 0.025–0.673 μm; one-way ANOVA, p = 0.1161, F = 2.198, N = 21, total df  = 20). The vast majority (90%) of synapse-associated polyribosomes located in dendritic shafts and spines was within 0.6 μm from the synapses. All polyribosomes located in dendritic spines were not farther than 0.940 μm away from the centers of synapses.

#### Density of polyribosomes located in dendritic spines

**Figure 2:**
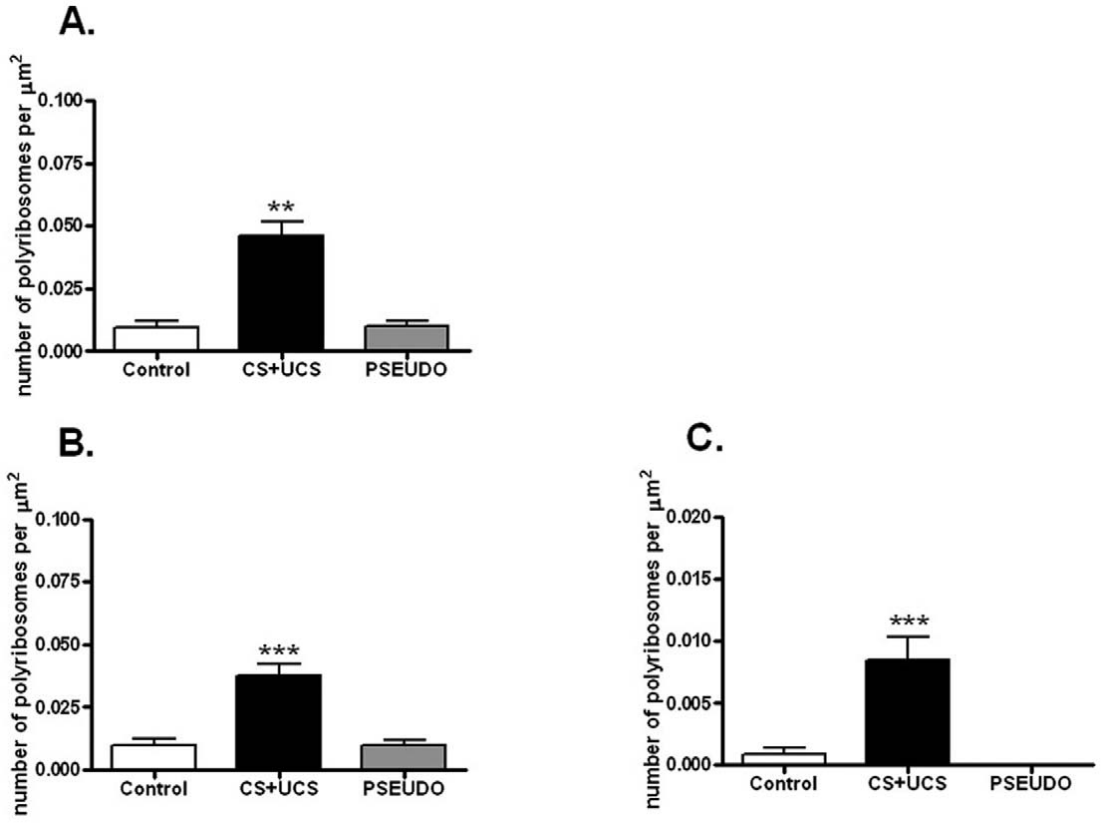
Density of polyribosomes in dendritic spines. Conditioning increases the density of polyribosomes located in spines and associated with excitatory (ANOVA, p<0.001) and inhibitory synapses (ANOVA, p<0.001). **A.** Total density of polyribosomes located in dendritic spines in: control, conditioned (CS+UCS) and pseudoconditioned (PSEUDO) group. **B.** Density of polyribosomes located in spines and associated with excitatory synapses. **C**. Density of polyribosomes located in spines and associated with inhibitory synapses. All graphs show means ± SD (**p<0.01, ***p<0.001).

The density of polyribosomes located in the dendritic spines increased more than fourfold after conditioning (control group: 0.010±0.006/μm^2^; conditioned group: 0.046±0.014/μm^2^; Kruskal-Wallis test, p = 0.0012, H = 13.39, N = 21, total df  = 20) but not after pseudoconditioning (0.010±0.006/μm^2^) (Fig. 2A). Whisker conditioning increased the density of polyribosomes located in spines and associated with excitatory synapses almost fourfold, from 0.010±0.007/μm^2^ in the control animals to 0.038±0.011/μm^2^ (one-way ANOVA, p<0.0001, F = 26.28, N = 21, total df  = 20) (Fig. 2B) and with inhibitory synapses even more, from 0.001±0.001/μm^2^ in the control animals to 0.009±0.005/μm^2^ (one-way ANOVA, p<0.0001, F = 17.10, N = 21, total df  = 20) in the conditioned ones (Fig. 2C).

#### Density of polyribosomes located in dendritic shafts

**Figure 3:**
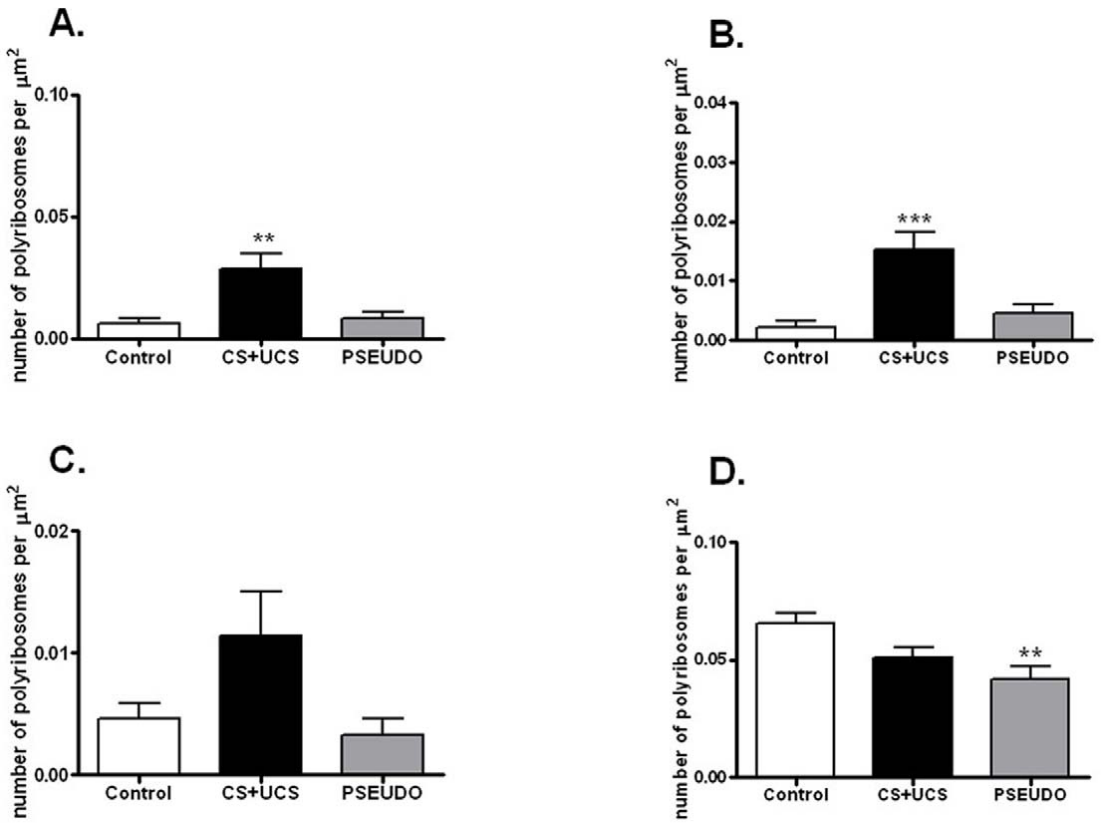
Density of polyribosomes in dendritic shafts. Conditioning induces an increase in the density of polyribosomes associated with inhibitory synapses and located in dendritic shafts (ANOVA, p<0.001), while pseudoconditioning decreases the density of polyribosomes not associated with synapses and located in dendritic shafts (ANOVA, p<0.01). **A.** Density of polyribosomes associated with synapses and located in dendritic shafts in: control, conditioned (CS+UCS) and pseudoconditioned (PSEUDO) group. **B.** Density of polyribosomes associated with inhibitory synapses and located in dendritic shafts. **C.** Density of polyribosomes associated with excitatory synapses and located in dendritic shafts. **D.** Density of polyribosomes not associated with synapses and located in dendritic shafts. All graphs show means ± SD (**p<0.01, *** p<0.001).

*Polyribosomes associated with synapses*: The conditioning induced a significant increase in the density of polyribosomes associated with synapses and located in dendritic shafts (control group: 0.006±0.004/μm^2^; conditioned group: 0.029±0.016/μm^2^; Kruskal-Wallis test, p = 0.0026, H = 11.89, N = 21, total df  = 20)(Fig. 3A). An increase was observed in the density of polyribosomes associated with presumptive inhibitory synapses (control group: 0.002±0.003/μm^2^; conditioned group: 0.015±0.008/μm^2^; one-way ANOVA, p = 0.0004, F = 12.48, N = 21, total df  = 20)(Fig. 3B). The density of polyribosomes associated with presumptive excitatory synapses did not significantly change either in the conditioned or in the pseudoconditioned animals, as compared with the control animals (control group: 0.005±0.003/μm^2^; conditioned group: 0.011±0.010/μm^2^; pseudoconditioned group: 0.003±0.004/μm^2^; Kruskal-Wallis test, N = 21, total df  = 20)(Fig. 3C).

*Polyribosomes not associated with synapses:* In the conditioned group, the mean density of polyribosomes not associated with synapses did not change (0.068±0.021/μm^2^), but in the pseudoconditioned group it was by ∼35% lower (0.049±0.014/μm^2^) than in the control group (0.076±0.009/μm^2^, one-way ANOVA, p = 0.0119, F = 5.719, N = 21, total df  = 20) (Fig. 3D).

### Location of polyribosomes in dendritic spines

Using 3D reconstructions of dendritic spines, we examined the effect of conditioning on distribution of polyribosomes in the spines. Polyribosomes were located in the head, neck or base of the dendritic spine. The base of spine was defined as the area within 150 nm of the neck's origin [12].

**Figure 4:**
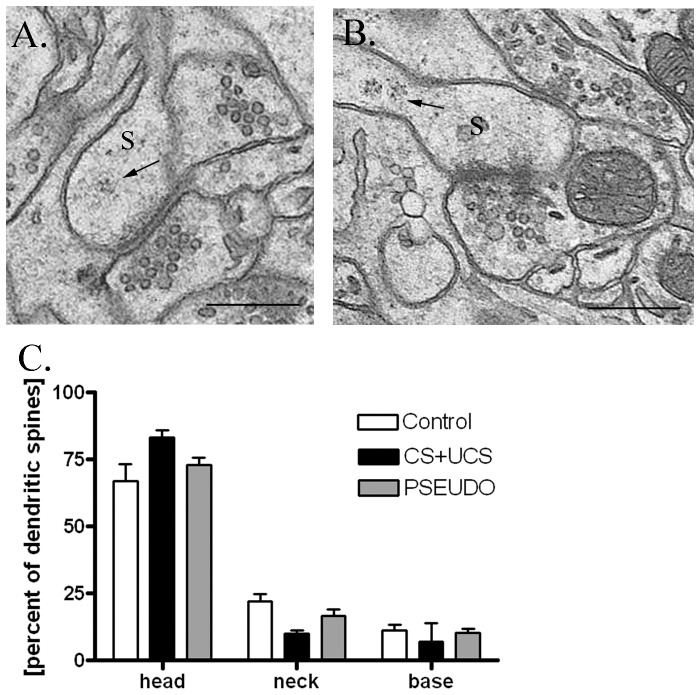
Location of polyribosomes in heads/necks/bases of dendritic spines. Conditioning and pseudoconditioning do not change the location of polyribosomes in the dendritic spines. **A.** Electron micrograph of B2 barrel hollow showing polyribosomes (arrow) located in the head of single-synapse spine (s). **B.** Electron micrograph of B2 barrel hollow showing polyribosomes (arrows) located in the neck of single-synapse spine (s). Scale bar: 0.5 μm. **C.** Location of polyribosomes in dendritic spines in: control, conditioned (CS+UCS) and pseudoconditioned (PSEUDO) group. Graph shows means ± SD.

Conditioning and pseudoconditioning did not significantly change the location of polyribosomes, as compared to that found in the control animals (chi square test, χ^2^ = 4.90, N = 3, df  = 2) (Fig. 4).

### Area of postsynaptic density (PSD)

**Figure 5:**
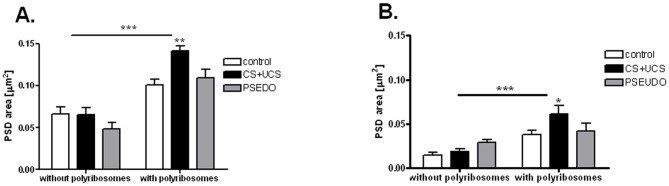
Postsynaptic density area. **A.** Excitatory synapses located on spines containing polyribosomes have larger postsynaptic density area than those on spines without polyribosomes (two-way ANOVA, p<0.0001) and the synapses enlarge during conditioning (ANOVA, p<0.01). Synapses on spines without polyribosomes do not significantly differ in size in the control, in the conditioned group (CS+UCS) and in the pseudoconditioned group (PSEUDO). Graph shows means ± SD (*p<0.05, *p<0.01, ***p<0.001). **B**. Inhibitory synapses located on spines containing polyribosomes have larger postsynaptic density area of than those located on spines without polyribosomes (two-way ANOVA, p<0.0001) and the synapses enlarge during conditioning (ANOVA, p<0.05). Synapses on spines without polyribosomes do not significantly differ in size in the control, in the conditioned group (CS+UCS) and in the pseudoconditioned group (PSEUDO).

Synapse-associated polyribosomes which increase in density after conditioning might produce proteins necessary for the enhancement of the synaptic response and increase in the synapse size. To test this hypothesis, we measured the area of postsynaptic density (PSD) of both, excitatory and inhibitory synapses. Since the thickness of PSD is quite uniform in the given synapse type, PSD area reflects also the synapse size. We observed a significant difference between the synapses on spines with and without polyribosomes (two-way ANOVA, N = 21) (Fig. 5). The synapses on the spines without polyribosomes had the same PSD area in the control (excitatory synapses: 0.066±0.021 μm^2^, inhibitory synapses: 0.015±0.007 μm^2^), in the conditioned animals (excitatory synapses: 0.065±0.021 μm^2^; inhibitory synapses: 0.019±0.007 μm^2^; two-way ANOVA, t = 0.0845, p>0.05, and t = 0.4048, p>0.05 for the two synapse types, respectively) and in the pseudoconditioned animals (excitatory synapses: 0.048±0.021 μm^2^; inhibitory synapses: 0.029±0.007 μm^2^; two-way ANOVA, t = 0.0480, p>0.05 and t = 1.346, p>0.05 for the two synapse types, respectively).

The synapses associated with polyribosomes in all groups had significantly larger PSD area, as compared to those not associated with polyribosomes (F = 72.29, p<0.0001 and F = 21.14, p<0.0001 for excitatory and inhibitory synapses, respectively). After conditioning PSD area of both excitatory and inhibitory synapses on spines with polyribosomes significantly increased (to 0.141±0.016 μm^2^ and to 0.061±0.029 μm^2^, respectively; two-way ANOVA, t = 3.384, p<0.01 and t = 2.665, p<0.05, respectively).

## Discussion

We have found that associative fear learning involving stimulation of whiskers significantly increases the density of polyribosomes associated with inhibitory and excitatory synapses on the dendritic spines and only with inhibitory synapses on the dendritic shafts of the barrels receiving the conditioned stimulus. Interestingly, conditioning did not affect the density of polyribosomes not associated with synapses in the dendritic shafts, suggesting a link between local protein synthesis and synaptic activity.

We used the pseudoconditioned group to test whether our finding was directly associated with the influence of fear learning or whether it resulted only from the application of two kinds of sensory stimuli. From our previous studies we know that pseudoconditioning does not change the functional cortical representation of the “trained” row of whiskers [16]. It also does not bring about the behavioral “minifreezing” effect, an indicator of CS-UCS association. The number of polyribosomes in the neuropil of the barrels of the pseudoconditioned animals was not significantly different when compared with the control group. Thus, the observed changes in the number of polyribosomes are linked to plastic modifications of the barrel cortex induced by conditioning.

In the previous study, we observed the formation of new symmetric (inhibitory) synapses at dendrites during conditioning. An increased concentration of GABA was found in the presynaptic terminals of these synapses. In addition, the number of double-synapse spines was higher after the learning procedure [20]. Therefore it seems likely that the polyribosomes located in double-synapse spines are associated with the experience-dependent formation of new inhibitory synapse at the spines. There is no data available in the literature on local translation of specific proteins of GABA-ergic inhibitory synapses (GABA receptor subunits, gephyrin, GIRK2 channel) or on the presence of mRNAs for these proteins in dendrites. Interestingly, mRNA for GABAa receptor delta subunit was found in dendrites and its content was increased upon mGluR stimulation [30]. Also, Racca et al. [31] reported that mRNA for GIRK2, inwardly rectifying potassium channel that mediates the inhibitory action of GABAb receptor, colocalized with Nova splicing proteins at the synapse. Hence, the increase in the size of inhibitory synapses located on spines containing polyribosomes in the conditioned animals suggests the involvement of polyribosomes in both synaptogenesis and potentiation of the synapses [12].

Since we have not observed an increase in the density of single-synapse spines after fear learning [20], the polyribosomes found in single-synapse spines are most likely involved in the modification of the existing excitatory synapses [14] rather than in building new ones. We found that the area of PSD in single-synapse spines was enlarged after conditioning. On the postsynaptic side, the size of the PSD is proportional to both the total number of ionotropic glutamate receptors and the AMPA to NMDA receptor ratio [32], [33], [34]. The coincident increase in polyribosome number and enlargement of PSD in the single-synapse spines suggests a potential involvement of local protein synthesis in enhancement of the strength of the excitatory synapses at the spines [23], [24].

A large pool of mRNAs was found in dendrites [35], [36], [37]. Some of the proteins encoded by these mRNAs could be synthesized in response to stimulation [36], [38]. They include a variety of different classes and some of them have been identified, such as the α subunit of calcium/calmodulin – dependent protein kinase II (CaMKII) [36], [39]. Some of the newly produced proteins may serve to insert glutamate receptors and rebuild the postsynaptic density scaffolding, to reorganize the chemoarchitecture of the potentiated synapses [23], [40]. Our previous studies provided some evidence for the changes of the glutamate receptors in our experimental model. In layer IV of the barrel cortex, within the plastic cortical representation of the whisker row used in the training, we observed an increase in expression of NMDA receptor NR2A subunit mRNA and protein [22]. We also found an increase in the level of PSD95, a locally synthesized scaffolding molecule [41], [42], in enriched postsynaptic membrane fraction of the barrel cortex [43]. PSD95 molecules are known to diffuse from spine to spine in the developing neocortex [44]. The conditioning-induced up-regulation of PSD95 can initially result from its transfer to potentiated spines from a diffuse cytoplasmic pool [45] but the long-term persistence of a higher PSD95 level suggests a subsequent local translation of PSD95 mRNA.

In conclusion, this study provides the first evidence that fear learning, which modifies cortical whisker representations, results in mobilization of organelles responsible for local protein synthesis in both inhibitory and excitatory synapses in the layer IV of the barrel cortex. This effect is accompanied by increased inhibitory synaptogenesis and enlargement of PSD area of polyribosome-associated excitatory and inhibitory synapses on spines. Remodeling of cortical circuits by excitatory and inhibitory interactions was observed at the electrophysiological level in other experimental models of learning, in which the neuronal excitability was found to be increased together with an enhancement of inhibitory effects [46], [47], [48], [49]. Similarly, in our experimental model, we have demonstrated that conditioning is followed not only by an increase in inhibitory efficiency [20], [21] but also by an enhanced responsiveness of the excitatory neurons [50]. Enhanced inhibition in layer IV produces feedforward inhibition which sharpens the tuning of excitatory barrel neurons [51], [52]. Mutual regulation of excitation and inhibition may prevent neurons in the cortical representation of the trained whiskers to become hypo- or hyperactive and to maintain the ability to produce action potentials in response to fluctuating inputs. It can also contribute to an increase in the selectivity to the “trained” input within the conditioned cortical representation and lead to improved perception of stimuli of a novel biological significance.

Our results show some elements of a mechanism responsible for an adjustment of excitatory-inhibitory balance during fear learning-dependent plasticity in the barrel cortex and present yet more evidence that selective regulation of dendritic spines is central to the mechanisms underlying the plasticity of the neuronal circuits.
